# Pea eggplant (*Solanum torvum* Swartz) is a source of plant food polyphenols with SARS-CoV inhibiting potential

**DOI:** 10.7717/peerj.14168

**Published:** 2022-11-29

**Authors:** Nisha Govender, Norazura Syazlin Zulkifli, Nurul Farhana Badrul Hisham, Nur Syatila Ab Ghani, Zeti-Azura Mohamed-Hussein

**Affiliations:** 1 Institute of Systems Biology (INBIOSIS), Universiti Kebangsaan Malaysia, Bangi, Selangor, Malaysia; 2 Infrastructure University Kuala Kumpur (IUKL), Kajang, Selangor, Malaysia; 3 Faculty of Science and Technology, Universiti Kebangsaan Malaysia, Bangi, Selangor, Malaysia

**Keywords:** COVID-19, Pea eggplant, Molecular docking, Molecular dynamics simulation, SARS CoV, Polyphenols

## Abstract

**Background:**

Pea eggplant (*Solanum torvum* Swartz) commonly known as turkey berry or **‘**terung pipit’ in Malay is a vegetable plant widely consumed by the local community in Malaysia. The shrub bears pea-like turkey berry fruits (TBFs), rich in phytochemicals of medicinal interest. The TBF phytochemicals hold a wide spectrum of pharmacological properties. In this study, the TBF phytochemicals’ potential inhibitory properties were evaluated against severe acute respiratory syndrome coronavirus 2 (SARS-CoV-2) of the Coronavirus disease 2019 (COVID-19). The TBF polyphenols were screened against SARS-CoV receptors *via* molecular docking and the best receptor-ligand complex was validated further by molecular dynamics (MD) simulation.

**Method:**

The SARS-CoV receptor structure files (viral structural components) were retrieved from the Protein Data Bank (PDB) database: membrane protein (PDB ID: 3I6G), main protease (PDB ID: 5RE4), and spike glycoproteins (PDB ID: 6VXX and 6VYB). The receptor binding pocket regions were identified by Discovery Studio (BIOVIA) for targeted docking with TBF polyphenols (genistin, kaempferol, mellein, rhoifolin and scutellarein). The ligand and SARS-CoV family receptor structure files were pre-processed using the AutoDock tools. Molecular docking was performed with the Lamarckian genetic algorithm using AutoDock Vina 4.2 software. The best pose (ligand-receptor complex) from the molecular docking analysis was selected based on the minimum binding energy (MBE) and extent of structural interactions, as indicated by BIOVIA visualization tool. The selected complex was validated by a 100 ns MD simulation run using the GROMACS software. The dynamic behaviour and stability of the receptor-ligand complex were evaluated by the root mean square displacement (RMSD), root mean square fluctuation (RMSF), radius of gyration (Rg), solvent accessible surface area (SASA), solvent accessible surface volume (SASV) and number of hydrogen bonds.

**Results:**

At RMSD = 0, the TBF polyphenols showed fairly strong physical interactions with SARS-CoV receptors under all possible combinations. The MBE of TBF polyphenol-bound SARS CoV complexes ranged from −4.6 to −8.3 kcal/mol. Analysis of the structural interactions showed the presence of hydrogen bonds, electrostatic and hydrophobic interactions between the receptor residues (RR) and ligands atoms. Based on the MBE values, the 3I6G-rhoifolin (MBE = −8.3 kcal/mol) and 5RE4-genistin (MBE = −7.6 kcal/mol) complexes were ranked with the least value. However, the latter showed a greater extent of interactions between the RRs and the ligand atoms and thus was further validated by MD simulation. The MD simulation parameters of the 5RE4-genistin complex over a 100 ns run indicated good structural stability with minimal flexibility within genistin binding pocket region. The findings suggest that *S. torvum* polyphenols hold good therapeutics potential in COVID-19 management.

## Introduction

In March 2020, the World Health Organisation (WHO) declared Coronavirus disease 2019 (COVID-19) caused by the severe acute respiratory syndrome coronavirus 2 (SARS-CoV-2), a pandemic. The contagious respiratory disease has been threatening public health with multiple waves of infections across the globe. As of 7^th^ June 2022, COVID-19 had recorded 500 million cases with 6.3 million deaths, worldwide (https://www.worldometers.info/coronavirus/). The disease symptoms extend from mild, severe and critically ill conditions; cough, sore throat, flu, systemic inflammatory response, innate and adaptive immune responses, T cell response and severe lymphopenia (King & Sprent, 2021).

SARS-CoV-2 belongs to the coronaviridae family and betacoronavirus genus of SARS-CoV and MERS-CoV human pathogens. The positive-sense virion has a single-strand RNA genome with two untranslated regions (UTRs) and 12 open reading frames (ORFs) encoding accessory proteins, non-structural proteins and structural proteins (Gordon et al., 2020). The structural proteins namely the spike (S), envelope (E), nucleocapsid (N) and membrane (M) protein encode 1,273 amino acid (aa), 75 aa, 419 aa and 222 aa, respectively. The ORF genes encode accessory proteins. The S protein (180 kDa) is composed of fusion peptide, heptad repeat 1, heptad repeat 2, intracellular domain, N-terminal unit subdomain 1, N-terminal unit subdomain 2, a transmembrane region and receptor-binding domain (RBD). The RBD mediates viral attachment to angiotensin-converting enzyme 2 (ACE2). Thereafter, the S protein is cleaved by host proteases. The S protein fuses into the host membrane before protein conformational changes take place during host cell entry (Lan et al., 2020; Jaimes et al., 2020). The E and M proteins are involved in virus packing and N coordinates the viral RNA binding and packaging (Mariano et al., 2020).

Two prominent clinical features observed in COVID-19 patients include active innate immune cell response and lymphocyte suppression (adaptive immunity). The first correlates to disease severity whilst the latter corresponds to fatality. In fatal cases, COVID-19 patients develop a dangerous immune response termed ‘cytokine storm’ (Mulchandani, Lyngdoh & Kakkar, 2021) whereby the immune system synthesizes excessive cytokines (immune cells) without control and damages the kidney, lungs and heart. These immune cells, known as the cytotoxic T cells, release toxic compounds in response to virus invasion (Berlin, Gullick & Martinez, 2020). During this event, the human immune system perceives a self-suicidal mode as excessive toxin production impairs organ functionality (Amor, Fernandez Blanco & Baker, 2020; Schreiber, 2020). Severe COVID-19 cases manifest fatal infection of the lower respiratory tract, pneumonia and multi-organ failures.

In various countries especially amongst the low- and middle-income countries (LMIC), poly- or single herbal plant species are deployed in the form of cocktail and decoction for COVID-19 prevention, palliative care and subsequent treatment; Kabasura Kudineer (India), Shuanghuanglian (China) and *Andrographis paniculate* (Thailand) (Usuzaki, Chiba & Shimoyama, 2021). Nutrient-dense food especially of plant origin promotes good health and protects the human body from diseases. Phytochemicals protect the human body from natural damages and reverse the existing damage along with damage-inducing activities for healing and disease prevention (Ali et al., 2022; Lee et al., 2021). The evolution of modern medicine is tightly linked to herbalism and thus, it is worth noting that a vast majority of modern medicines are plant-derived compounds (phytochemicals). To date, the dietary phytochemicals had received greater recognition as pharmaceuticals. Extending further, plant foods are equally important in human diets.

**Figure 1:**
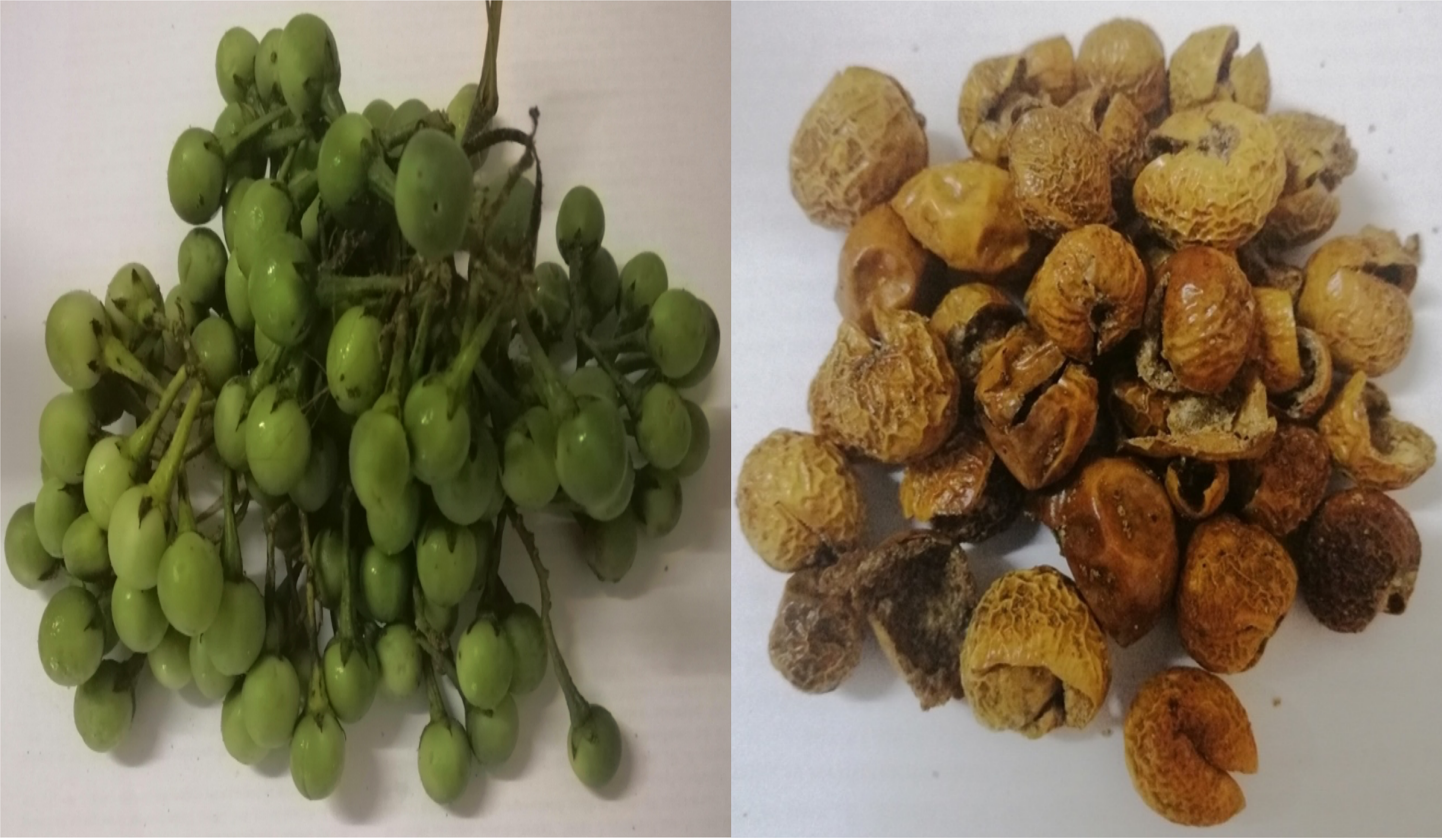
The pea eggplant (*Solanum torvum*) fresh (left) and sun-dried (right) fruits.

The number of studies on the potential anti-COVID-19 phytochemicals had accelerated rapidly in recent times. Fruits and vegetables are excellent sources of phenolic compounds with high antioxidant activity (Agregan et al., 2021; Singh et al., 2009). Eggplants from the Solanaceae family represent a group of nutritious vegetable fruits enriched with bioactive compounds (Bidaramali, Akhtar & Das, 2020). They inherit abundant polyphenols such as phenolic acids, anthocyanins and antioxidants. Polyphenols carry health-beneficial properties such as antidiabetic, antibacterial, anti-fungal, and anti-inflammatory, and thus, are exploited in the pharmaceutical industry and innovative functional food development (Chah, Muko & Oboegbulem, 2000; Singh et al., 2009; Agregan et al., 2021). Amongst the Solanaceae family, turkey berry (*Solanum torvum* Swartz) fruits (TBF) (Fig. 1) have shown the highest total phenolic content (TPC) at 190.8 mg GAE/g (solvent = 50% ethanol concentration) in comparison to its close relatives; *S. ferrugineum* (31.41 mg GAE/g), *S. melongena* (16.97 mg GAE/g), *S. betaceum* (24.74 mg GAE/g) and *S. retroflexum* (92.07 mg GAE/g) (Weremfo et al., 2022). In others, TBF has been reported to contain alkaloids, flavonoids, saponins, tannins, isoflavonoid sulfate, steroidal glycosides (Arthan et al., 2002) and glycosides (Sivapriya & Srinivas, 2007).

The growing open-source information on SARS-CoV receptors had paved an avenue for rapid screening and identification of potent phytochemicals from a diverse collection of natural products (Cherrak, Merzouk & Mokhtari-Soulimane, 2020; Cortés-García et al. 2020; Das et al., 2021). Molecular docking and molecular dynamics (MD) simulation has become an important tool to evaluate the inhibitory actions of phytochemicals against viral receptors (Gangwal et al., 2013; Aissouq et al., 2020). In this study, three different SARS-CoV receptor targets were selected based on their structural involvement in the various mechanisms of SARS-CoV pathogenesis and life cycle: spike protein, main protease and membrane protein (Rahman et al. 2020; Rothan & Byrareddy 2020; Walls et al. 2020). The spike protein is a type 1 transmembrane S glycoprotein distributed on the surface of SARS-CoV-2. It plays a key role in coordinating the viral entry into host cells (ACE2). The main protease (M^pro^) facilitates proteolysis of large polyproteins, which then are orderly packaged into new virions. The membrane protein modulates the maturation and retention processes of the virion assembly (Boson et al., 2021). The inhibitory potentials of pea eggplant polyphenols against SARS-CoV proteins are investigated to shed meaningful insights into *S. torvum* therapeutic potential in COVID-19 management (De Boer et al., 2022).

## Materials and Methods

### Identification and pre-processing of SARS-CoV protein targets

The 3-dimensional structures of SARS-CoV family proteins were retrieved from the Protein Data Bank (https://www.rsb.org/). The receptor information is presented in Table 1. Each receptor was pre-processed using AutoDock tools 1.5.6 (*Trott & Olson, 2010)* as follows: (i) add polar hydrogen atoms, (ii) add Kollman charges, (iii) eliminate the water molecules and (iv) eliminate heteroatoms (Berman et al., 2000). The binding pocket region (x, y and z spatial coordinates) of each receptor was identified using Biovia Discovery Studio (2021) (https://discover.3ds.com). The processed clean receptor files were subjected to molecular docking analysis. For ligand-bound native receptor structures, the discarded ligand was designated as a positive control in subsequent molecular docking analysis.

**Table 1:** Structural description of SARS-CoV protein targets obtained from RCSB Protein Data bank.

PDB ID	Protein description	Resolution	Strcuture of the protein
6VYB	SARS-CoV-2 spike ectodomain structure (open state)	3.20 Å	Spike glycoprotein
6VXX	Structure of the SARS-CoV-2 spike glycoprotein (closed state)	2.80 Å	Spike glycoprotein
5RE4	Crystal structure of SARS-CoV-2 main protease in complex with Z1129283193	1.88 Å	Main protease
3I6G	Newly identified epitope Mn2 from SARS-CoV M protein complexed with HLA-A*0201	2.20 Å	Membrane protein

### Identification and pre-processing of *Solanum torvum* polyphenols

The following *S. torvum* polyphenols (PubChem ID expressed in parenthesis) were selected as candidate ligands and the corresponding 2-dimensional structures were obtained from PubChem Compound Database (https://pubchem.ncbi.nlm.nih.gov): genistin (CID: 5281377), kaempferol (CID: 5280863), mellein (CID: 28516), rhoifolin (CID: 5282150) and scutellarein (5281697). The native files were converted into protein format using PyMOL 2.5 (https://pymol.org). The ligands were pre-processed to remove heteroatom (if present), assign torsion and add Gasteiger partial charges using AutoDock Tools 1.5.6 (Trott & Olson, 2010). The structure information of the candidate ligands is provided in Table 2.

**Table 2:** The pea eggplant (*Solanum torvum*) polyphenol two-dimensional structures.

Ligand	2D Structure
Genistin	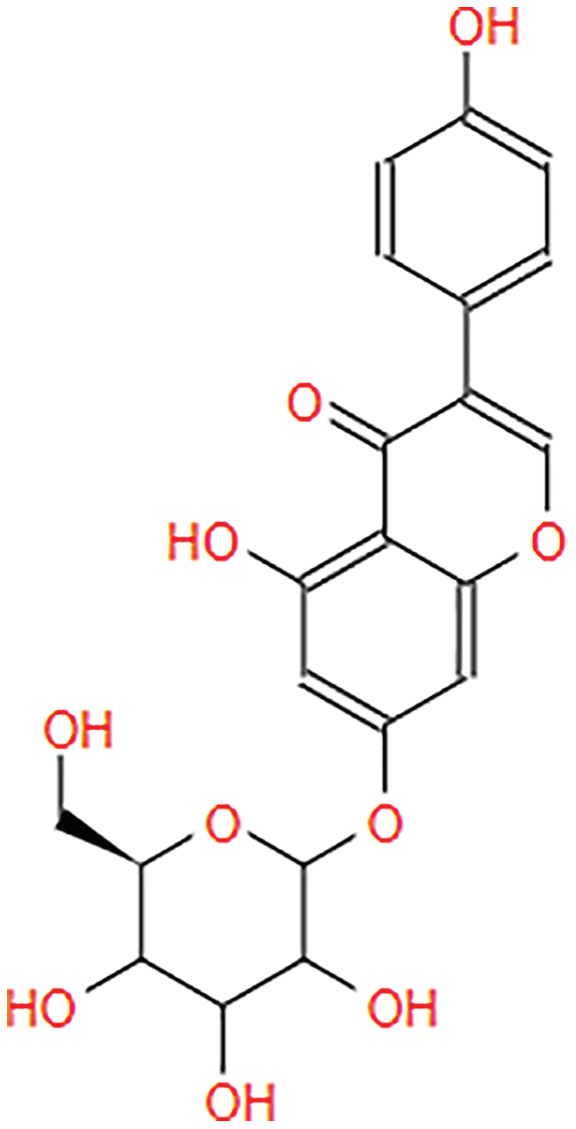
Kaempferol	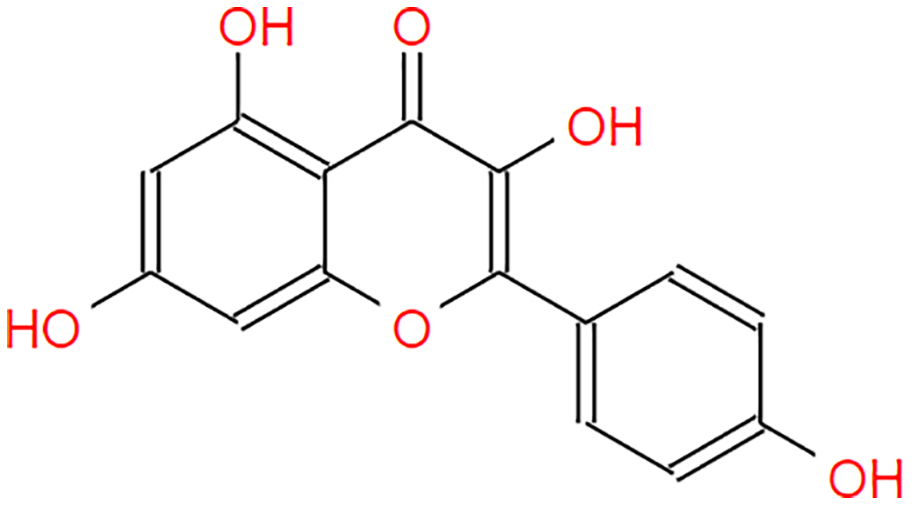
Mellein	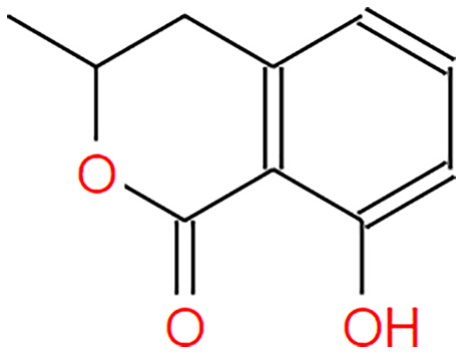
Rhoifolin	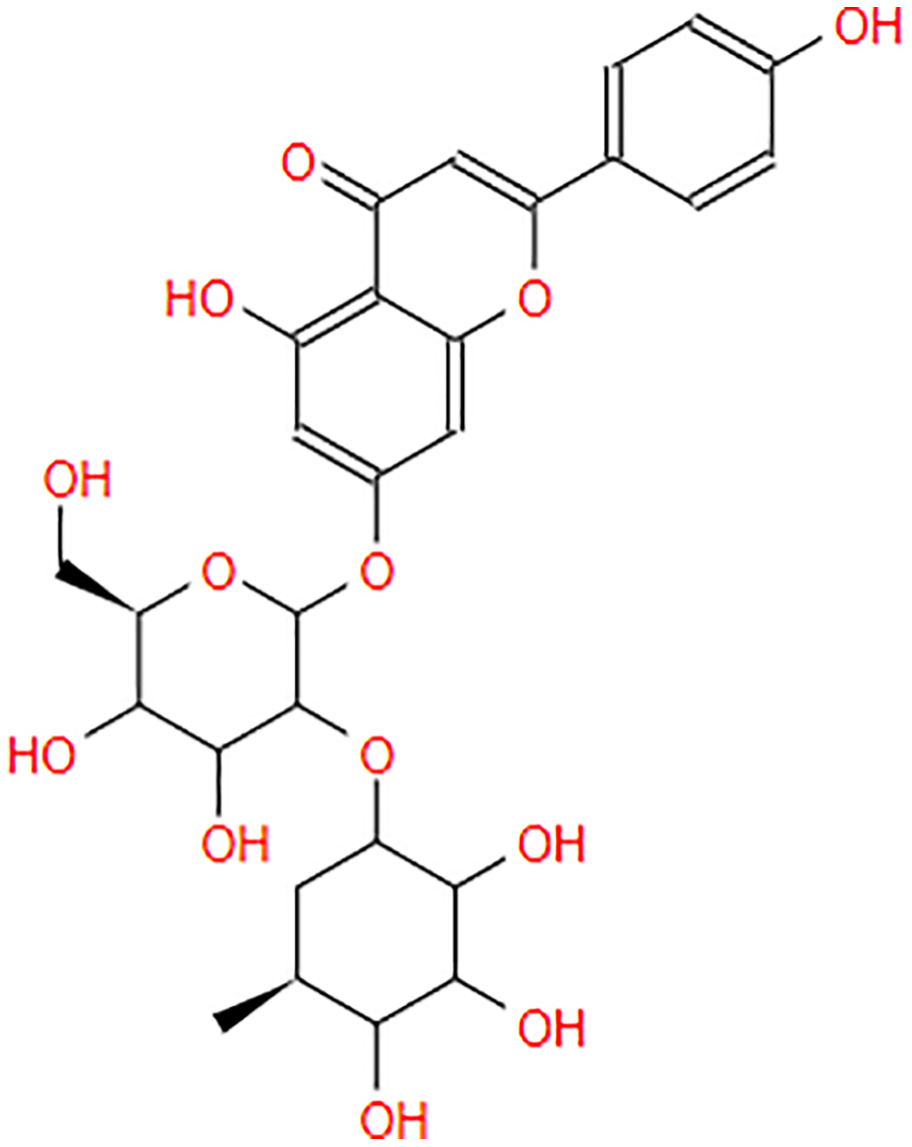
Scutellarein	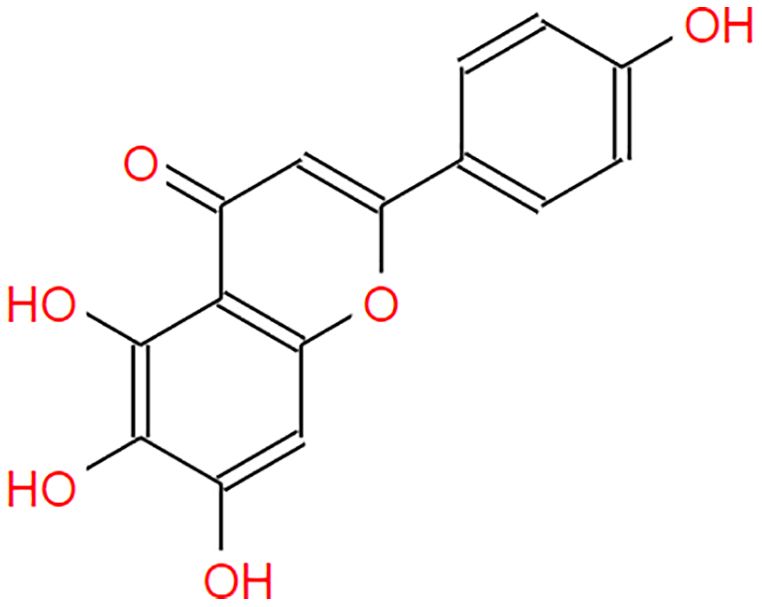

### Drug-likeness and oral-bioavailability analysis of *Solanum torvum* polyphenols

The *S. torvum* polyphenols (as indicated in the previous section) were subjected to drug-likeness and oral bioavailability analysis using the SwissADME online tool (http://www.swissadme.ch/) (Daina, Michielin & Zoete, 2017).

### Molecular docking analysis

The receptor-ligand molecular docking was performed using Autodock Vina (Trott & Olson, 2010). A grid box for each receptor was set based on the x, y and z dimensions; coordinates corresponding to the binding pockets region of the receptor (configuration file). Each target ligand was docked against the receptor under all possible pair-wise (receptor-ligand) combinations. The co-crystallized native ligand in each receptor was used as the reference standard. In receptors without a native ligand, azithromycin, a drug used to inhibit SARS-COV-2 (Kaddoura et al., 2020) was employed as the standard molecule (Table 3). Following molecular docking, the receptor-ligand complex with the best confirmation was selected for molecular dynamics (MD) simulation based on the minimum binding energy (MBE) values, the number of hydrogen bonds, hydrophobic bonds and other weak interactions involved in stabilizing the complex. The receptor-ligand complex visualization was performed using BIOVIA Discovery Studio (2021) (https://discover.3ds.com) and PyMOL 2.5 (https://pymol.org).

**Table 3:** Minimum binding energy (kcal/mol) of the pea eggplant (*Solanum torvum)* polyphenol-bound SARS-CoV family receptor complexes at RMSD = 0. The polyphenols (genistin, kaempferol, mellein, rhoifolin and scutellarein) are ligands complexed with SARS-CoV receptors (PDB ID: 3I6G, 5RE4, 6VXX and 6VYB) under all possible combinations.

Receptor (PDB ID)	Binding pocket region	Ligand	Minimum binding affinity (kcal/mol)
3I6G	x = 2.09852	genistin	−7.3
	y = 7.48756	kaempferol	−6.5
	z = 40.02637	mellein	−5.5
		rhoifolin	−8.3
		scutellarein	−6.3
		azithromycin^+^	−11.3
5RE4	x = 7.11073	genistin	−7.6
	y = 0.24564	kaempferol	−6.1
	z = 20.21082	mellein	−4.9
		rhoifolin	−6.5
		scutellarein	−5.8
		N-(4-methylpyridin-3-yl)acetamide^+^	−4.9
6VXX	x = 181.11671	genistin	−5.7
	y = 233.11289	kaempferol	−5.6
	z = 243.26782	mullein	−5.0
		rhoifolin	−6.6
		scutellarein	−5.3
		2-acetamido-2-deoxy-beta-D-glucopyranose^+^	−4.2
6VYB	x = 177.69014	genistin	−5.7
	y = 231.70471	kaempferol	−5.7
	z = 240.69318	mellein	−4.6
		rhoifolin	−6.0
		scutellarein	−5.3
		2-acetamido-2-deoxy beta-D-glucopyranose^+^	

**Note:**

+ Positive control.

### Molecular dynamics (MD) simulation

The MD simulation was performed using GROMACS software on a Ubuntu system. The receptor and ligand topology files were prepared manually. The receptor topology was prepared using GROMACS built-in tool, pdb2gmx. The CHARMM General Force Field (CGenFF) program was used to generate the ligand topology according to Vanommeslaeghe et al. (2010). The CHARMM36 all-atom force field (Feb 2021) was retrieved from the ParamChem project (http://mackerell.umaryland.edu). Each ligand-receptor complex was solvated in a triclinic box using the transferable intermolecular potential with a three-points (TIP3P) water model. The force-field parameters were generated by CGenFF server (https://www.paramchem.org/) under the CGenFF program. The maximum number of minimization steps was set at 50,000 and the energy step size at 0.01. The ‘nsteps’ of number of particles, volume and temperature (NVT) and number of particles, pressure and temperature (NPT) ensemble equilibration was set at 50,000 ns (100 ps). The temperature was set at 300 K and the MD simulation was run for 100 ns under constant pressure (NPT ensemble) and long-range electrostatic interactions generated using the Particle Mesh Ewald (PME) algorithm. All MD trajectories were analyzed using GROMACS built-in tools. The *grmsd package* measured root-mean-square-deviation (RMSD) variation of the protein backbone, the *grmsf package* measured the overall root-mean-square-fluctuation (RMSF) at the atomic positions of the protein C backbone and the *gyrate and g h bond* package evaluated the radius of gyration (Rg) and the number of hydrogen bonds of receptor-ligand complex, respectively. The *gmx sasa* package computed the change of solvent accessible surface areas (SASA) and solvent accessible surface volume (SASV). All plots were prepared using Microsoft Excel.

## Results

**Figure 2:**
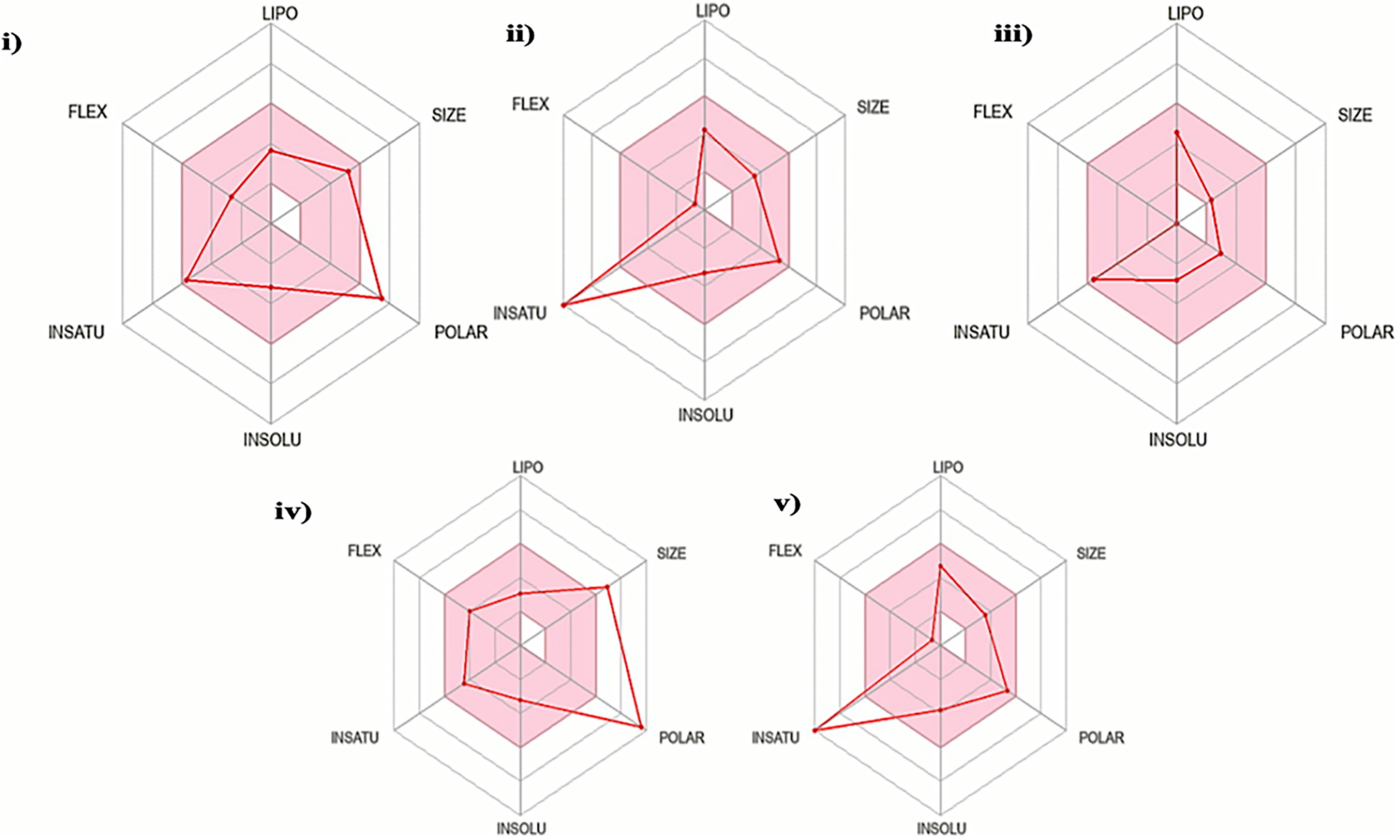
Characterization of *Solanum torvum* polyphenols’ oral bioavailability by SwissADME (http://www.swissadme.ch/), chemoinformatics tool: (i) genistin, (ii) kaempferol, (iii) mullein, (iv) rhoifoilin and (v) scutellarein. The pink region within the RADAR (hexagon) denotes area with optimum physiochemical properties. Each physiochemical property evaluated is abbreviated as following along with the corresponding optimum values: LIPO; Lipophilicity; −0.7-to-+5.0 (xlogP3), SIZE; 500 g/mol, POLAR; 20–130 Å2 (TPSA), INSOLU; 0–6 (ESOL, log S), INSATU; 0.5–1 (Fraction Csp3) and FLEX; number of rotatable bonds <9.

The oral bioavailability analysis of *S. torvum* polyphenols revealed the following physicochemical properties: LIPO; lipophilicity, SIZE; size expressed in molecular weight, POLAR; polarity, INSOLU; insolubility, INSATU; unsaturation and FLEX; flexibility. The polyphenol properties corresponding to oral bioavailability fall within an acceptable range, with at least four matches out of the six properties. The lipophilicity, flexibility and insolubility of genistin, kaempferol, mullein, rhoifoilin and scutellarein were in an ideal range, as denoted by the connecting red dots which fall within the pink region of the RADAR (Fig. 2). The lipophilicity, as measured by xlogP3 range from −0.16 to 2.66. Flexibility, measured by the number of rotatable bonds ranges from zero to six. Insolubility, measured by ESOL range from −2.82 to −3.79. The size (molecular weight) of all the *S. torvum* polyphenols lies within an optimum range of <500 g/mol except rhoifolin (578.52 g/mol). Both genistin (170.05 Å²) and rhoifolin (228.97 Å²) deviate from the recommended range for polarity while the rest of the S. *torvum* polyphenols meet the standard requirement at 46.53-to-111.13 Å² (Supplemental 1).

According to Lipinski’s rule of five (RO5), a drug-like compound must meet at least 3/4 of the following criteria (one violation is allowed): (i) molecular weight ≤500 Da, (ii) hydrogen bond donor ≤5, (iii) hydrogen bond acceptor ≤10 and (iv) (log P) ≤5 (Lipinski, 2004). In this study, all the *S. torvum* polyphenols agreed with RO5 except rhoifolin (three violations). A similar pattern of results was obtained using the Ghose criteria. The bioavailability score for all the *S. torvum* polyphenols was 0.55 except rhoifolin (Supplemental 1). Molecular docking analysis predicted the minimum binding energy (MBE) values and structural interactions between selected *S. torvum* polyphenols and SARS-CoV receptors. For 5RE4, 6VXX and 6VYB receptors, the native ligands present in the unmodified structure files were designated as positive control reference molecules. The molecular docking of each receptor with its target ligands was performed in parallel with the corresponding positive controls, as follows: (i) 5RE4-N-(4-methylpyridin-3-yl)acetamide, (ii) 6VXX-2-acetamido-2-deoxy-beta-D-glucopyranose and (iii) 6VYB-2-acetamido-2-deoxy-beta-D-glucopyranose. Since 3I6G was free from native ligands, the azithromycin COVID-19 drug was assigned as the positive control ligand.

**Figure 3:**
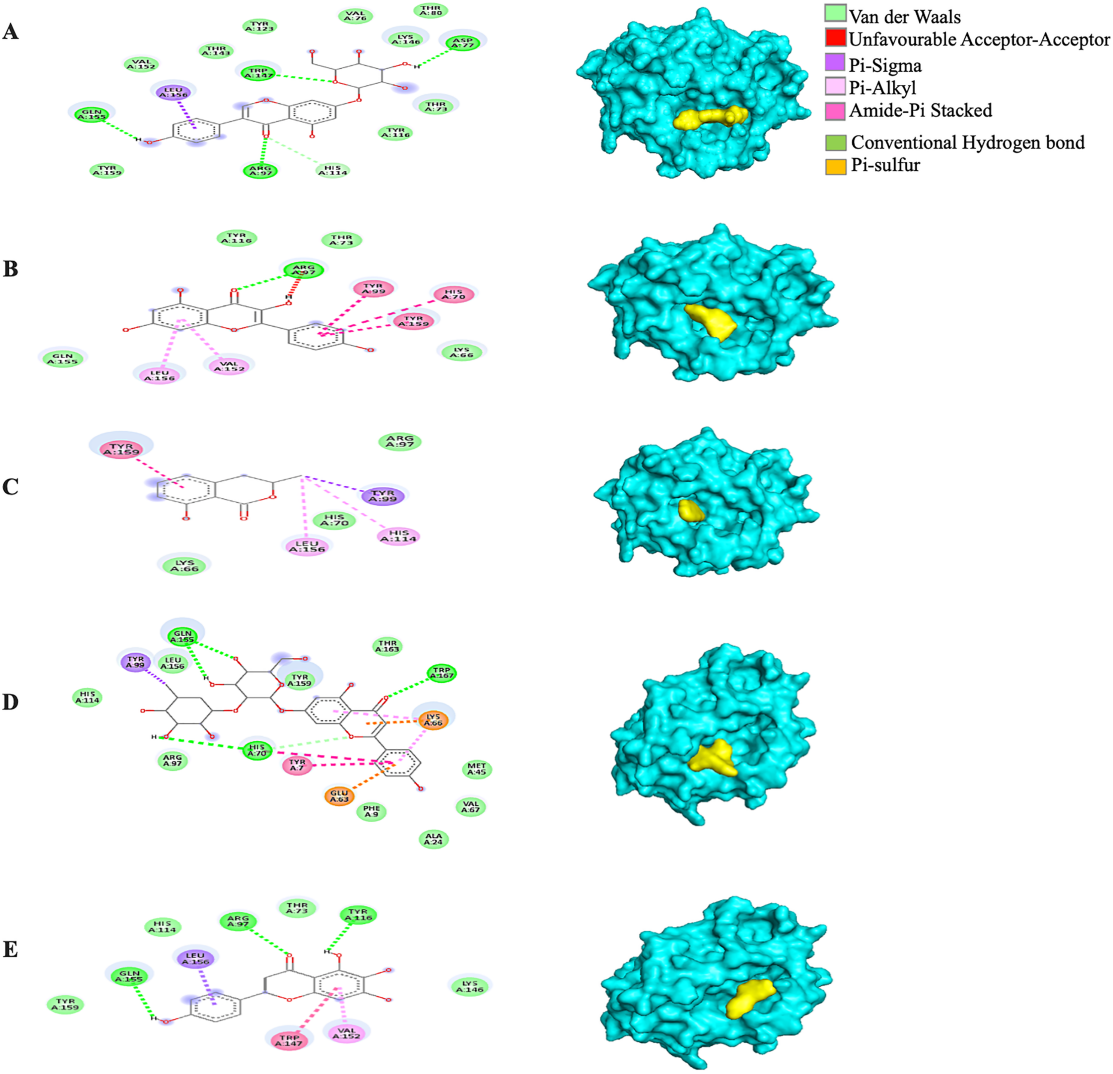
The *Solanum torvum* polyphenol-bound SARS-CoV receptor (PDB ID: 3I6G) complexes. Different alphabets represent the unique ligand-bound 3I6G complex: (A) genistin, (B) kaempferol, (C) mellein, (D) rhoifolin and (E) scutellarein. All ligand-receptor complexes are obtained at RMSD = 0. The right column shows the 3-D surface representation and the left column shows 2-D visualization of the interactions between the ligand atom and receptor residues. The yellow and blue globular surface representations are ligand and receptor, respectively (right column).

Generally, under all possible receptor-ligand pair-wise combinations, the MBE ranged from −4.6 to −8.3 kcal/mol. In 3I6G-bound ligand complexes, the MBE ranged from −5.5 to −8.3 kcal/mol with various extents of hydrogen bonds, hydrophobic interactions and electrostatic interactions. However, the 3I6G-kaempferol and 3I6G-mellein complexes showed an absence of hydrogen bonds. The 3I6G-rhoifolin complex (−8.3 kcal/mol) showed the least MBE followed by the 3I6G-genistin complex at MBE = −7.3 kcal/mol. The highest number of interactions were observed in 3I6G-bound complexes: three hydrogen bonds receptor residues (RR): ASP77, TRP147, GLN155, five hydrophobic interactions (RR: TYR159, TYR123, VAL76, THR80, TYR116) and two electrostatic interactions (RR: ARG97, LYS146) between the receptor residues and the ligand. Interestingly, the electrostatic interactions among the 3I6G-bound ligand complexes were consistently formed at the ARG and LYS (Fig. 3).

**Figure 4:**
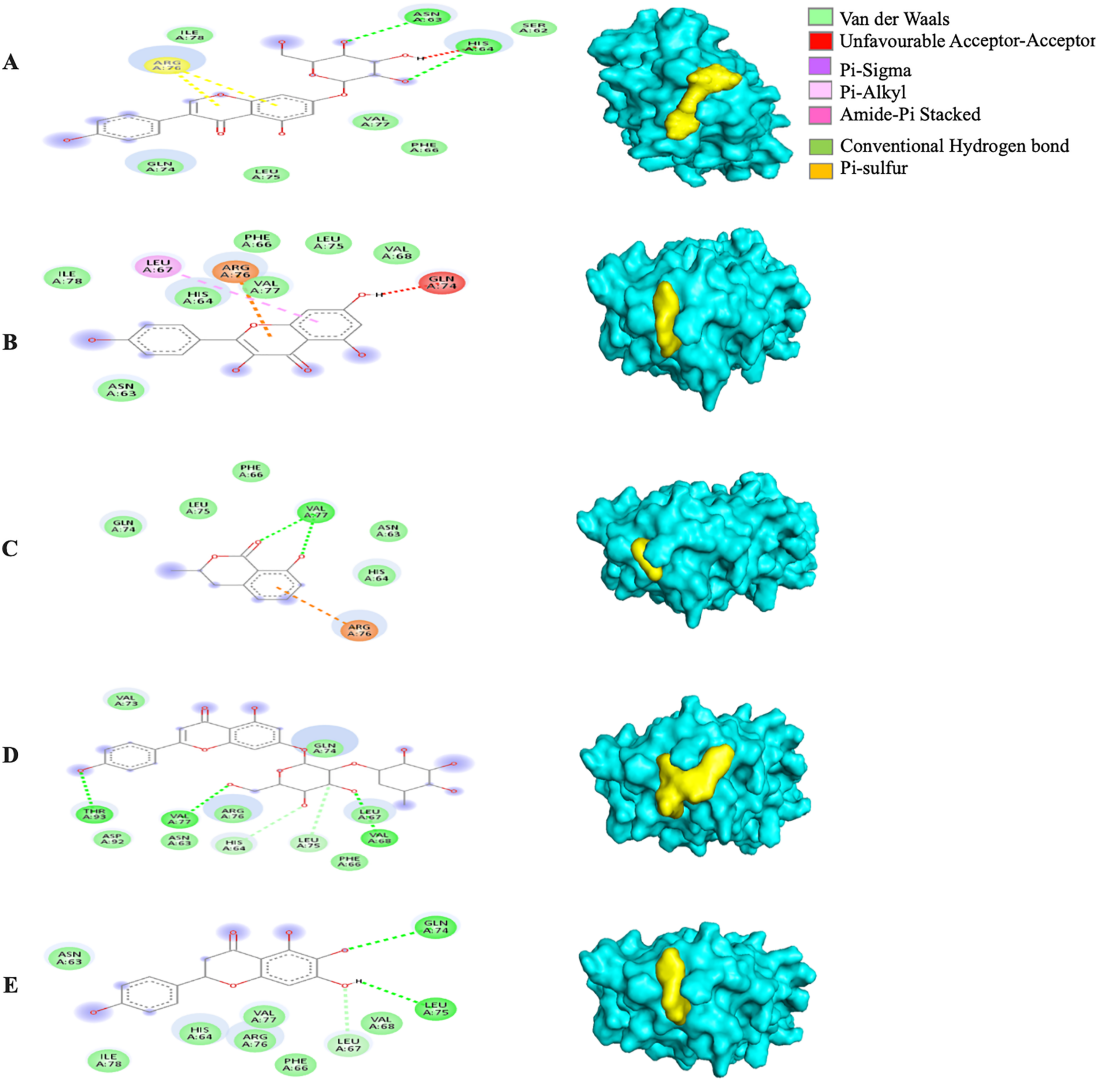
The *Solanum torvum* polyphenol-bound SARS-CoV receptor (PDB ID: 5RE4) complexes. Different alphabets represent the unique ligand-bound 5RE4 complex: (A) genistin, (B) kaempferol, (C) mellein, (D) rhoifolin and (E) scutellarein. All ligand-receptor complexes are obtained at RMSD = 0. The right column shows the 3-D surface representation and the left column shows 2-D visualization of the interactions between the ligand atom and receptor residue. The yellow and blue globular surface representations are ligand and receptor, respectively (right column).

Among the 5RE4-bound ligand complexes, the 5RE4-genistin complex showed the least MBE at −7.6 kcal/mol followed by 5RE4-rhoifolin complex and 5RE4-kaempferol complex at −6.5 and −6.1 kcal/mol, respectively. All 5RE4-bound ligand complexes showed at least three or more hydrophobic interactions at various RR (SER62, ILE78, PHE66, LEU75, ILE78, VAL68, ASP92, ASN63) and an electrostatic interaction at ARG76 only. The number of hydrogen bonds was highest in the 5RE4-rhoifolin complex (RR: THR93, VAL77, VAL68) while the rest showed 1–2 hydrogen bonds only. The 5RE4-kaempferol complex showed no occurrence of hydrogen bonds (Fig. 4).

**Figure 5:**
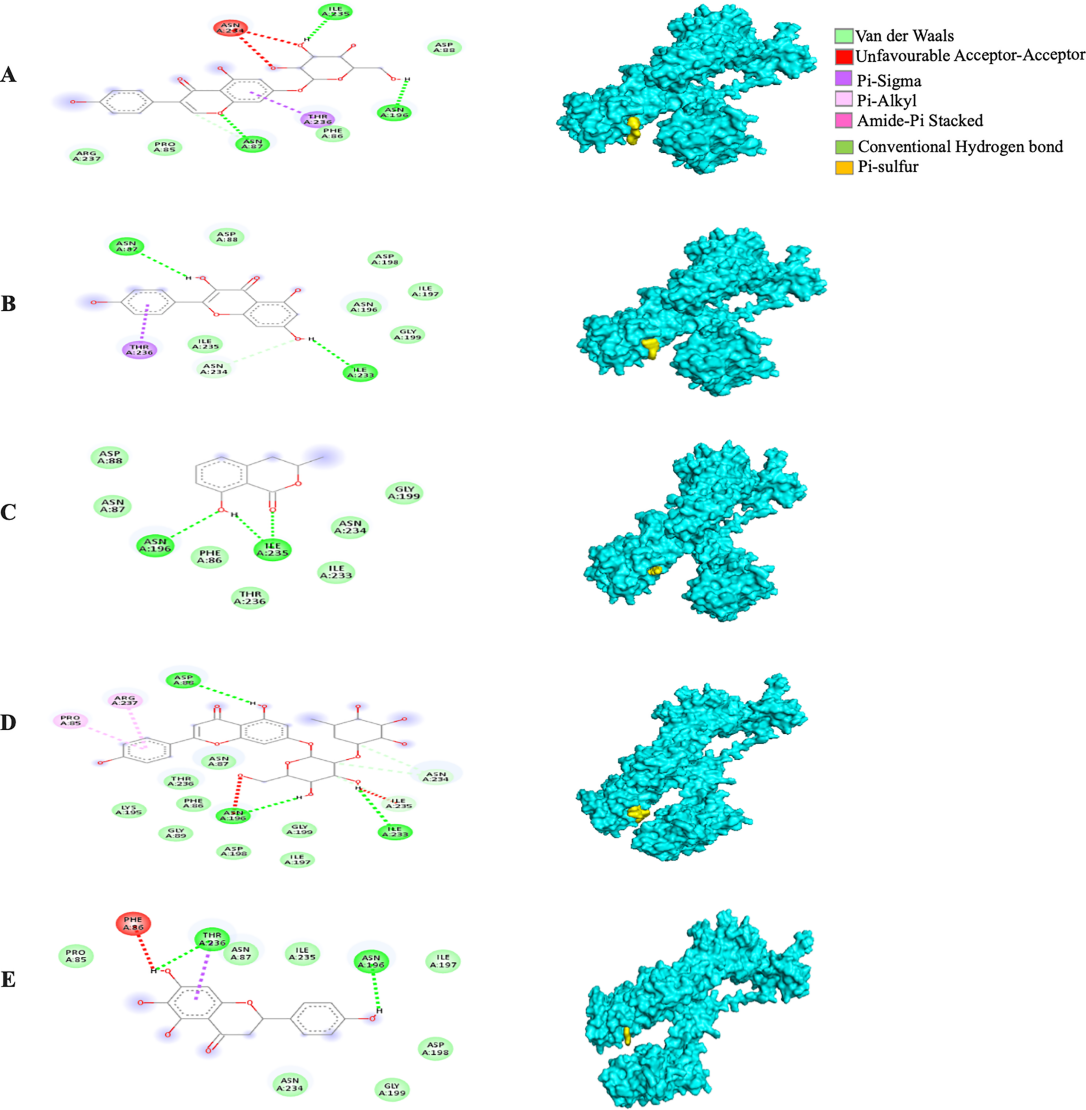
The *Solanum torvum* polyphenol-bound SARS-CoV receptor (PDB ID: 6VXX) complexes. Different alphabets represent the unique ligand-bound 6VXX complex: (A) genistin, (B) kaempferol, (C) mellein, (D) rhoifolin and (E) scutellarein. All ligand-receptor complexes are obtained at RMSD = 0. The right column shows the 3-D surface representation and the left column shows 2-D visualization of the interactions between the ligand atom and receptor residues. The yellow and blue globular surface representations are ligand and receptor, respectively (right column).

The MBE of the 6VXX-bound ligand complexes ranged at −5 to −6.6 kcal/mol and at least two hydrogen bonds were present. The 6VXX-rhoifolin complex showed the least MBE at −6.6 kcal/mol alongside the greatest number of structural interactions: three hydrogen bonds (RR: ASP88, ASN196, ILE233), five hydrophobic (RR: PHE86, GLY89, ASP198, ILE197, GLY199) and three electrostatic (RR: ARG237, LYS195) interactions. With a total of five hydrophobic attractions, the 6VXX-mellein and 6VXX-rhoifolin complexes showed the highest number of hydrophobic attractions with two common sites at residue PHE86 and GLY199. The electrostatic interactions were absent in all the 6VXX-bound ligand complexes except for 6VXX-rhoifolin and 6VXX-genistin complex; the electrostatic attraction was formed at ARG237 residue (Fig. 5).

**Figure 6:**
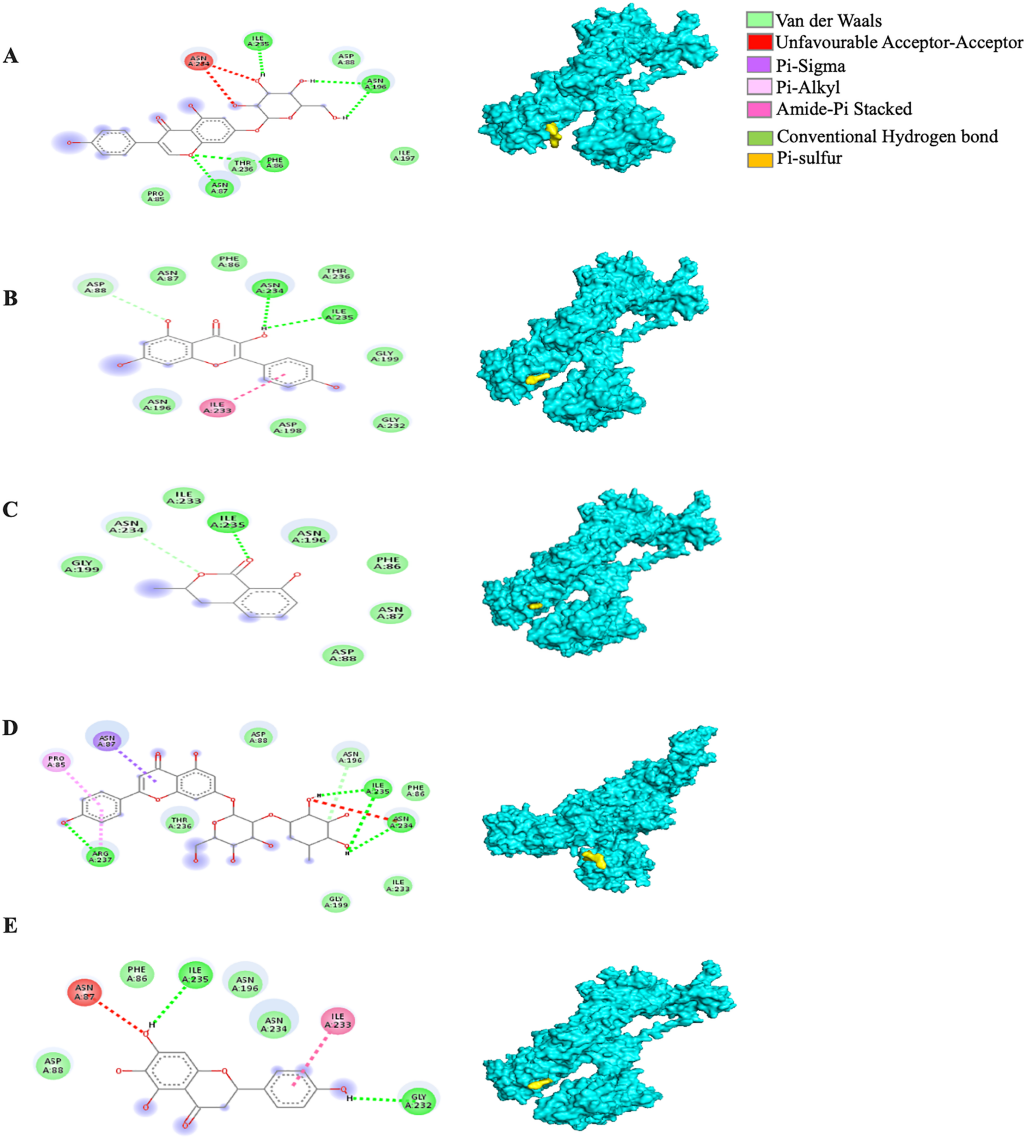
The *Solanum torvum* polyphenol-bound SARS-CoV receptor (PDB ID: 6VYB) complexes. Different alphabets represent the unique ligand-bound 6VYB complex: (A) genistin, (B) kaempferol, (C) mellein, (D) rhoifolin and (E) scutellarein. All ligand-receptor complexes are obtained at RMSD = 0. The right column shows the 3-D surface representation and the left column shows 2-D visualization of the interactions between the ligand atom and receptor residues. The yellow and blue globular surface representations are ligand and receptor, respectively (right column).

The MBE of the 6VYB-bound ligand complexes ranged from −4.6 to −6.0 kcal/mol. The 6VYB-rhoifolin complex showed the least MBE at −6.0 kcal/mol followed by 6VYB-genistin and 6VYB-kaempferol complexes at MBE = −5.7 kcal/mol. All 6VYB-bound ligand complexes were held by hydrogen bonds and hydrophobic interactions. Generally, the 6VYB-bound ligand complexes formed a hydrogen bond at ILE235, RR. The 6VYB-genistin complex showed the highest number of hydrogen bonds at the ILE235, ASN196, ASN87 and PHE86, RR. The 6VYB-kaempferol complex was held by five hydrophobic interactions at the following RRs: PHE86, THR236, GLY199, GLY232 and ASP198. All 6VYB-bound ligand complexes showed an absence of electrostatic interactions except the 6VYB-rhoifolin complex (Fig. 6).

**Figure 7:**
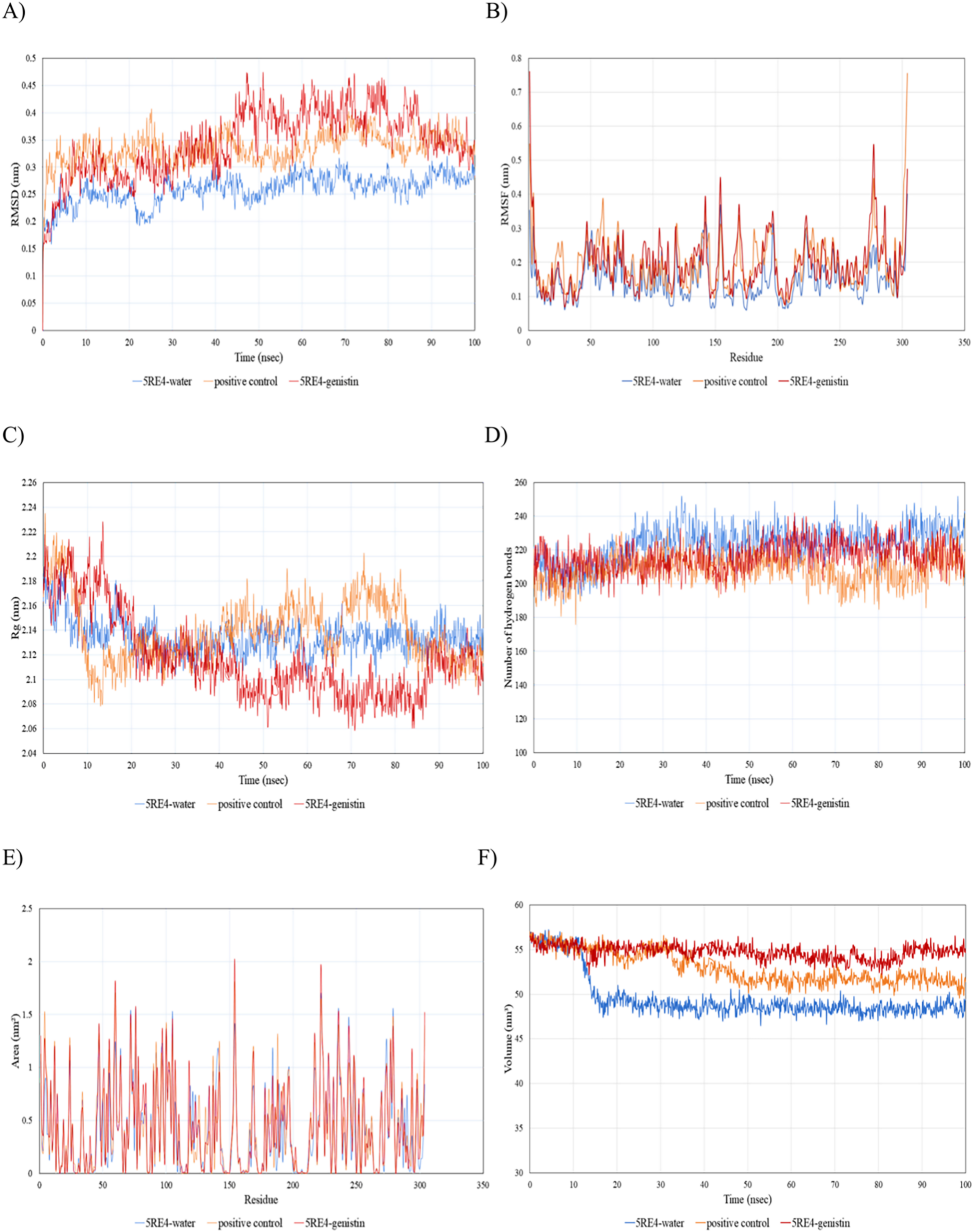
The trajectory analysis of the 5RE4-genistin complex under a 100 ns molecular dynamics simulation run. Positive control is represented by 5RE4 complexed with a native ligand, N-(4-methylpyridin-3-yl)acetamide. The 5RE4-water complex represents ligand free complex. (A) Complex root-mean-square-deviation (RMSD) of the complex and individual receptor molecule. (B) Complex root-mean-square-fluctuation (RMSF). (C) Complex radius of gyration (Rg). (D) Complex number of hydrogen bonds. (E) Complex solvent-accessible-surface area (SASA) and (F) Complex solvent-accessible-volume (SAV).

Since the 5RE4-genistin complex showed the least MBE with a fairly good number of structural interactions, the MD simulation analysis was carried out for further validation. The trajectory analysis of the 5RE4-genistin complex was evaluated over a 100 ns MD simulation run. The trajectory analysis of the 5RE4-water complex and 5RE4-N-(4-methylpyridin-3-yl)acetamide (positive control) were plotted along the 5RE4-genistin complex. The root mean square deviation (RMSD) of the receptor (5RE4) initially increased from 0.15–0.25 (15 nsec), and then collapse throughout the next 15 nsec before plunging up at 0.35–0.45 nm, fluctuation (40–100 nsec). The RMSD values of the 5RE4-genistin complex informed the deviation extent of the receptor-ligand complex against a reference structure (Schreiner et al., 2012). With a higher RMSD value, the stability of the receptor-ligand complex lowers. Conversely, lower RMSD values depict significant stability. At RMSD = 0.15–0.45, the 5RE4-genistin complex shows adequate genistin accommodation with 5RE4 binding pocket region. The 5RE4-genistin complex RMSD value range was slightly higher than the positive control (5RE4--N-(4-methylpyridin-3-yl)acetamide) and 5RE4-water complexes (Fig. 7A).

The root mean square fluctuation (RMSF) of 5RE4-genistin complex, 5RE4-N-(4-methylpyridin-3-yl) acetamide complex and 5RE4-water complex displayed a similar pattern, however, the 5RE4-water complex showed the least RMSF fluctuation range. The RMSF parameter sheds insights on the complex binding site stability. The flexibility criterion measures the contribution of the receptor’s individual residues to the receptor-ligand structural fluctuations. A large average deviation of receptor residue from the reference position corresponds to mobility increment (Benson & Daggett, 2013). The RMSF of 5RE4-genistin complex ranged at 0.05–0.55 nm, slightly close to the positive control (5RE4-N-(4-methylpyridin-3-yl) acetamide) complex at RMSF = 0.05–0.4 nm (Fig. 7B).

Radius of gyration (Rg) corresponds to the mass-weighted RMSD of a group of atoms relative to their common mass center (Likic et al., 2005). The lower the Rg values, the higher is the compactness or the global stability of the receptor-ligand complex. The Rg values of 5RE4-genistin complex ranged in between 2.06 to 2.24. During the first 20 nsec of the MD run, the Rg values of the 5RE4-genistin complex declined before levelling at 40–90 nsec. The Rg values reached plateau at an average value of 2.08–2.12 nm. The 5RE4-genistin complex fluctuations suggest good compactness and stability of genistin within the 5RE4 active site and was comparable to the positive control (5RE4-N-(4-methylpyridin-3-yl)acetamide) and 5RE4-water complexes (Fig. 7C).

The number of hydrogen bonds significantly contributes to the conformational changes and stability of the receptor-ligand complexes (Humphrey, Dalke & Schulten, 1996). The relative frequency of hydrogen interactions in 5RE4-genistin, 5RE4-water and 5RE4-N-(4-methylpyridin-3-yl)acetamide complexes ranged from 180–240. The 5RE4-genistin showed a fairly stable number of hydrogen bonds at 200–240 (Fig. 7D). The solvent-accessible-surface area (SASA) refers to the surface area of the receptor that is in contact with the residing solvent. The 5RE4-genistin, 5RE4-water and 5RE4-N-(4-methylpyridin-3-yl)acetamide solvent-accessible-surface area (SASA) of the receptor binding regions displayed a similar pattern throughout the 100 ns run. The SASA assesses the molecular surface area to solvent molecules, providing vital information on the extent of receptor-to-solvent interaction (Pirolli et al., 2014). Higher SASA corresponds to lower stability and a lower SASA depicts *vice-versa*. The solvent-accessible volume (SAV) is much refined to include the effect of solvents on the protein’s interior (Lazaridis & Karplus, 1999). In this study, the 5RE4-genistin SASA displayed a similar trend to SAV. The 5RE4-water complex showed the smallest SASA and SAV ranges at <1.5 nm^2^ and 45–50 nm^2^ respectively, whereas 5RE4-genistin showed the highest fluctuation range (SASA; <2 nm^2^ and SAV; 50–60 nm^2^).

## Discussion

Since the emergence of COVID-19 in late 2019, vaccination has emerged as the utmost viable strategy to ease the rate of infection and disease severity. Vaccines were rolled out in late 2020 with continuous inspection of the systemic and local side effects and effectiveness following administration (Menni et al., 2021). On the other hand, a wide variety of natural herbal medicines and dietary bioactive compounds previously recognized for their antiviral, anti-inflammatory, immune-regulatory and organ protective properties (Srivastava & Saxena, 2020) were re-purposed for COVID-19 treatment and management. In most low and middle-income countries (LMIC), natural herbal medicines are utilized for COVID-19 prevention and treatment without clear scientific evidence due to their low cost, easy availability and low toxicity nature (Usuzaki, Chiba & Shimoyama, 2021; Jantan et al., 2022).

The Japanese “Foods for Specific Health Uses” (FOSHU) criteria define functional food as food that is consumed as part of a normal diet and holds targeted health benefits (Ohama, Ikeda & Moriyama, 2019). Stretching beyond basic nutrition, plant foods are bestowed with a wealth of medicinally potent bioactive compounds (phytochemicals). Specific plant food phytochemicals such as polyphenols, alkaloids and terpenoids are evident to delay the onset of diseases (cancer and cardiovascular diseases), enhance the immune response against infectious diseases and prevent chronic diseases (Chen & chen, 2013; Chauhan, Yadav & Quraishi, 2021). From a pharmacological viewpoint, the broad chemical diversity of plant food phytochemicals renders a challenging screening procedure in identifying pathogen inhibitors. With the advent of computational tools, plant food phytochemicals are screened rapidly to evaluate the mechanistic activities between the target phytochemical and receptor *via* computational approaches: molecular docking, molecular dynamics simulation and bioactivity prediction (Kushwaha et al., 2021; Sajid Jamal, Alharbi & Ahmad, 2022; Xu et al., 2020). More so, in time of the COVID-19 pandemic, numerous underexploited and indigenous plant food phytochemicals especially polyphenols were subjected to robust computational studies; *Pandanus conoideus* Lamk flavonoid compounds (Umar, 2021), *Bridelia retusa* (Umar et al., 2022) and others (Abdul-Hammed et al., 2021).

Pea eggplant (*S. torvum*) from the Solanaceae family is a vegetable plant abundantly distributed in tropical regions, especially within the Southeast Asian region. The shrub grows vertically with many branches and reaches up to 3 meters (Gandhi, Ignacimuthu & Paulraj, 2011). The tiny round-shape edible fruits are light green when young, and turn shiny yellowish-green when ripe (Fig. 6). In Malaysia, the fruits are either eaten fresh as a side dish or incorporated into sambal and curries. In terms of nutritional properties, *S. torvum* is a rich reservoir of alkaloids, flavones and lignans (Jayakumar & Murugan, 2016). Therapeutic bioactive compounds present in *S. torvum* fruits are mainly polyphenols; mullein, quercetin, myricetin glucosides, kaempferol 3-O-glucosyl-rhamnosyl-galactoside, genistein, rhoifolin, nepetin and scutellarein (Senizza et al., 2021). Apart of *S. torvum*, closely related members of the Solanum species such as the *S. erianthum* (potato tree) are also rich in phytochemicals such as lignans, sesquiterpenes monoterpenes, alkaloids (solasodine, solverbascine, solanocardinol, solamarine) and fatty acids (Peng et al., 2017). In this study, five different polyphenols were selected based on their natural abundance reported by a previous metabolomics study: genistein (isoflavonoid), rhoifolin, kaempferol and scutellarein are abundantly occurring flavonols in *S. torvum* and so does mullein (hydroxycoumarin) (Senizza et al., 2021).

In this study, SARS-CoV receptors involved in the various stages of SARS-CoV pathogenesis were selected: (1) surface spike (S) protein (PDB ID: 6VXX and 6VYB); modulates the viral entry and fusion into the host cell membrane, (2) main protease-replicase polyproteins (PDB ID: 5RE4); mediates virus morphogenesis and assembly and (3) membrane protein (PDBD ID: 3I6G); involved in viral gene expression and replication and facilitates the proteolytic processing of replicase polyproteins (Thomas, 2020; Ullrich & Nitsche, 2020; Michel et al., 2020). The target receptor structures showed a good resolution at 1.88–3.2 Å (Table 1).

Five different polyphenols (ligands: genistin, kaempferol, mellein, rhoifolin and scutellarein) docked with SARS CoV family proteins under all possible combinations and the corresponding MBE of the complexes ranged at −4.6 to −8.3 kcal/mol. The MBE values were comparable to other similar studies on phytochemicals and SARS CoV-2 receptor complexes (Forli et al., 2016). Previous *in silico* findings reported on alicin, gingerol, epicatechin-gallate, catechin, curcumin, oleuropein, apigenin-7-glucoside, naringenin, nelfinavir, lopinavir, kaempferol, quercetin, luteolin-7-glucoside, demethoxycurcumin complexed with SARS-CoV-2 spike protein (PDB ID: 6LU7) ranged from −4.03 to −7.6 kcal/mol (Khaerunnisa et al., 2020; Mulu et al., 2020). Others, such as flavonoids from honey and propolis complexed with the RNA binding domain of SARS-CoV-2 nucleocapsid phosphoprotein (PDB ID: 6VYO) showed MBE = −7.2 to −10.1 kcal/mol (Ali & Kunugi, 2021). Previous *in silico* study showed that genistin inhibits SAS-CoV-2 Mpro (PDBID: 6LU7) at MBE = −7.6 kcal/mol (Harisna et al., 2021).

The receptor-ligand interactions were comprised of covalent bonds and non-covalent bonds such as salt bridges (ionic), hydrogen bonds, ring interaction, van der Waals forces and hydrophobic interactions. These interactions cumulatively drive the protein folding and confirmation and contribute to ligand binding stability onto the receptor binding pocket regions. Hydrophobic interactions are the predominant contributors to the stability of proteins. Hydrogen bonding supports protein stability to a lesser extent compared to hydrophobic interactions. Hydrophobic binding is a key determinant of folding configuration equilibria in many native proteins (Pace et al., 2011). The electrostatic interactions are associated with the binding affinity, structure, stability, and biological reactivity of proteins and nucleic acids (Sharp, 2006).

In general, all the ligand-bound complexes displayed a similar trend across the different receptors. Amongst all the receptor-ligand complexes investigated in this study, the rhoifolin-bound receptor complexes showed relatively good MBE, and the number of hydrogen bonds, hydrophobic interactions, and electrostatic interactions. The number of hydrophobic interactions was greatest in the 6VXX-rhoifolin complex with MBE = −6.6 kcal/mol (Table 1). Likewise, the 5RE4-genistin complex (MBE = −7.6 kcal/mol) showed a good extent of interactions between the RRs and ligand atoms. At least one hydrophobic interaction was present in all the ligand-receptor complexes (Figs. 2–5). The MD simulation observed the conformational trajectory of the 5RE4-genistin complex over 100 ns. At 0.05–0.4 nm, the RMSF trajectory fluctuated less and thus, explains the low flexibility and good stability of the bonds form between the RRs and ligand atoms. With less distortion, the binding regions are well structured within the 5RE4-genistin complex. The radius of gyration of the 5RE4-genistin complex was fairly low and thus, suggests good binding stability of the complex under a folded confirmation. The binding of genistin with 5RE4 did not induce apparent structural changes in the complex. The number of hydrogen bonds is a critical determinant of the binding strength of the 5RE4-genistin complex. The number of hydrogen bonds in the 5RE4-genistin complex was nearing close to the unbound 5RE4-water complex, suggesting good conformational stability.

Based on previous findings, genistin, kaempferol, mellein, rhoifolin and scutellarein have shown antiviral properties at a clinical scale on various viral pathogens. Genistin has been proven to exhibit antiviral activity against Herpes B Virus (LeCher et al., 2019) while kaempferol showed antiviral druggability against the 3a channel protein of coronavirus (Schwarz et al., 2014). On the other hand, mellein has shown antiviral activity against influenza and Herpes simplex viruses (Zgórniak-Nowosielska et al., 1991) and rhoifolin inhibit the SARS-CoV 3CL protease (Russo et al., 2020). Scutellarein was demonstrated to carry inhibitory activities against the SARS coronavirus helicase (Yu et al., 2012).

## Conclusions

Since the emergence of COVID-19, there has been a burst of computational studies, massively screening natural products in search of lead compounds in drug development. The significant interactions between the receptor binding residue and the inhibitor molecule are measured by various thermodynamic and dynamic properties. Similar computational tools and pipelines are also employed in functional food development, ultimately to inform the functional values of metabolites naturally enriched in dietary vegetables and fruits. Herein, turkey berry fruits (TBF), an excellent source of dietary polyphenols were selected for *in silico* screening against SARS-CoV family receptors. Although the inhibitory values were not on par with the required criteria set in drug discovery, the results showed satisfactory mechanistic inhibitory actions of TBF polyphenols, especially genistin against COVID-19. The *S. torvum* fruit is an established vegetable plant within the Southeast Asian region. The phytochemical (genistin, kaempferol, mullein, rhiofolin and scutellarein) enriched food plant showed a good minimum binding energy against SARS-CoV receptors. Further validation by molecular dynamics (MD) simulation showed that the 5RE4-genistin complex is relatively stable over a 100 ns MD simulation run. These polyphenols are inherently available in the pea eggplant and thus, can be deployed in functional food development. However, further experimental-level validations which includes *in vitro* and *in vivo* analyses are required in innovative TBF-based functional food development for COVID-19.

## Supplemental Information

10.7717/peerj.14168/supp-1 Supplemental Information 1 *Solanum torvum* polyphenols: Bioavailability and drug-likeness analysis.

10.7717/peerj.14168/supp-2 Supplemental Information 2 Molecular dynamics simulation trajectory analysis: Raw data.

## Additional Information and Declarations

### Data Availability

The following information was supplied regarding data availability: The raw data are available in the Supplemental Files.
