# Supplementary material for: Pea eggplant (Solanum torvum Swartz) is a source of plant food polyphenols with SARS-CoV inhibiting potential
Source: PeerJ. 2022 Nov 29;10:e14168. doi: 10.7717/peerj.14168 (PMC9744172; doi:10.7717/peerj.14168)
Supplement: Supplemental Information 1 [file peerj-10-14168-s001.docx]

Supplementary 1: *Solanum torvum* polyphenols: Bioavailability and drug-likeness analysis

|  | Genistin | Kaempferol | Mellein | Rhoifolin | Scutellarein |
| --- | --- | --- | --- | --- | --- |
| **Physiochemical Properties** |  |  |  |  |  |
| Molecular Weight | 432.38 g/mol | 286.24 g/mol | 178.18 g/mol | 578.52 g/mol | 286.24 g/mol |
| Heavy Atoms | 31 | 21 | 13 | 41 | 21 |
| Aromatic Heavy Atoms | 16 | 16 | 6 | 16 | 16 |
| Fraction Csp3 | 0.29 | 0.00 | 0.30 | 0.44 | 0.00 |
| Rotatable Bonds | 4 | 1 | 0 | 6 | 1 |
| H-bond Acceptors | 10 | 6 | 3 | 14 | 6 |
| H-bond Donors | 6 | 4 | 1 | 8 | 4 |
| Molar Refractivity | 106.11 | 76.01 | 47.40 | 137.33 | 76.01 |
| TPSA | 170.05 Å² | 111.13 Å² | 46.53 Å² | 228.97 Å² | 111.13 Å² |
| **Lipophilicity** |  |  |  |  |  |
| iLOGP | 2.11 | 1.70 | 1.96 | 2.07 | 2.08 |
| XLOGP3 | 0.86 | 1.90 | 2.44 | -0.16 | 2.66 |
| WLOGP | 0.05 | 2.28 | 1.49 | -1.10 | 2.28 |
| MLOGP | -1.61 | -0.03 | 1.55 | -2.96 | -0.03 |
| SILICOS-IT | 0.35 | 2.03 | 1.96 | -1.17 | 2.03 |
| Consensus Log | 0.35 | 1.58 | 1.88 | -0.66 | 1.81 |
| **Water Solubility** |  |  |  |  |  |
| ESOL | -3.18 | -3.31 | -2.82 | -3.32 | -3.79 |
| Ali | -4.01 | -3.86 | -3.06 | -4.19 | -4.65 |
| SILICOS-IT | -2.69 | -3.82 | -2.44 | -1.48 | -3.82 |
| **Pharmacokinetics** |  |  |  |  |  |
| GI Absorption | Low | High | High | Low | High |
| BBB permeant | No | No | Yes | No | No |
| P-gp Subtrate | No | No | No | Yes | No |
| CYP1A2 Inhibitor | No | Yes | Yes | No | Yes |
| CYP2C19 Inhibitor | No | No | No | No | No |
| CYP2C9 Inhibitor | No | No | No | No | Yes |
| CYP2D6 Inhibitor | No | Yes | No | No | Yes |
| Skin Permeation | No | -6.70 cm/s | -5.65 cm/s | -9.94 cm/s | -6.16 cm/s |
| **Drug Likeness** |  |  |  |  |  |
| Lipinski | Yes;1 violation | Yes;0 violation | Yes; 0 violation | No;3 violation | Yes;0 violation |
| Ghose | Yes | Yes | Yes | No;4 violation | Yes |
| Veber | No;1 violation | Yes | Yes | No;1 violation | Yes |
| Egan | No;1 violation | Yes | Yes | No;1 violation | Yes |
| Muegge | No;2 violation | Yes | No;1 violation | No;3 violation | Yes |
| Bioavailability Score | 0.55 | 0.55 | 0.55 | 0.17 | 0.55 |
| **Medicinal Chemistry** |  |  |  |  |  |
| PAINS | 0 alert | 0 alert | 0 alert | 0 alert | 1 alert; catechol A |
| Brenk | 0 alert | 0 alert | 0 alert | 0 alert | 1 alert; catechol |
| Leadlikeness | No;1 violation | Yes | No; 1 violation | No;1 violation | Yes |
| Synthetic Accessibility | 5.12 | 3.14 | 2.62 | 6.33 | 3.04 |
